# Rhinolith Misdiagnosed as Fungal Mucin

**DOI:** 10.7759/cureus.46648

**Published:** 2023-10-07

**Authors:** Nuha Alrayes, Abdulrahman Alhumaizi, Alanoud Alomair, Rehab Simsim

**Affiliations:** 1 Otolaryngology-Head and Neck Surgery, King Abdullah bin Abdulaziz University Hospital, Riyadh, SAU; 2 Clinical Sciences, Princess Nourah bint Abdulrahman University, Riyadh, SAU

**Keywords:** nasal sinus tumor, nasal mass, fungal sinusitis, nasal foreign body, rhinolith

## Abstract

Foreign body insertion inside the nose is not uncommon in pediatric age groups. It can pass unnoticed by parents, sometimes underdiagnosed or incompletely removed by a clinician. In another scenario, it may be incidentally discovered by imaging during dental workups commonly. This foreign body acts like a nidus for a rhinolith, as it gets calcified over years and becomes like a stone, causing unilateral nasal symptoms. Herein, we present a case of a young female with a rhinolith mistaken for fungal mud. We aim to emphasize this rare clinical condition that, if left unperceived, may lead to complications including, but not limited to, sinusitis, pressure necrosis to the surrounding structure causing septal perforation, or naso-palatal fistula.

## Introduction

Rhinolithiasis is an uncommon condition seen in otolaryngology practice [1]. It is formed by calcification and mineralization around a nidus [1] that could be either endogenous or exogenous [2]. The clinical suspicion to diagnose rhinolith increases in young patients with unilateral nasal symptoms. Nonetheless, rhinolith can be found at any age. Usually, patients do not recall foreign body insertion into the nose [3]. This case report aims to increase awareness of rhinolithiasis in clinical practice and point out the importance of considering other diseases that may share similar radiological features of calcification, such as fungal sinusitis, as well as some benign and malignant nasal tumors.

## Case presentation

A 20-year-old healthy female presented to the otolaryngology clinic complaining of left-sided nasal blockage for nine months associated with unilateral nasal drainage. However, the patient denied hyposmia, facial pressure, facial edema, postnasal drip, headache, and nose bleeding. She also denies inserting a foreign body in her nose or having previous nasal trauma. Her family history was unremarkable for fungal rhinosinusitis or sinonasal malignancy.

**Figure 1:**
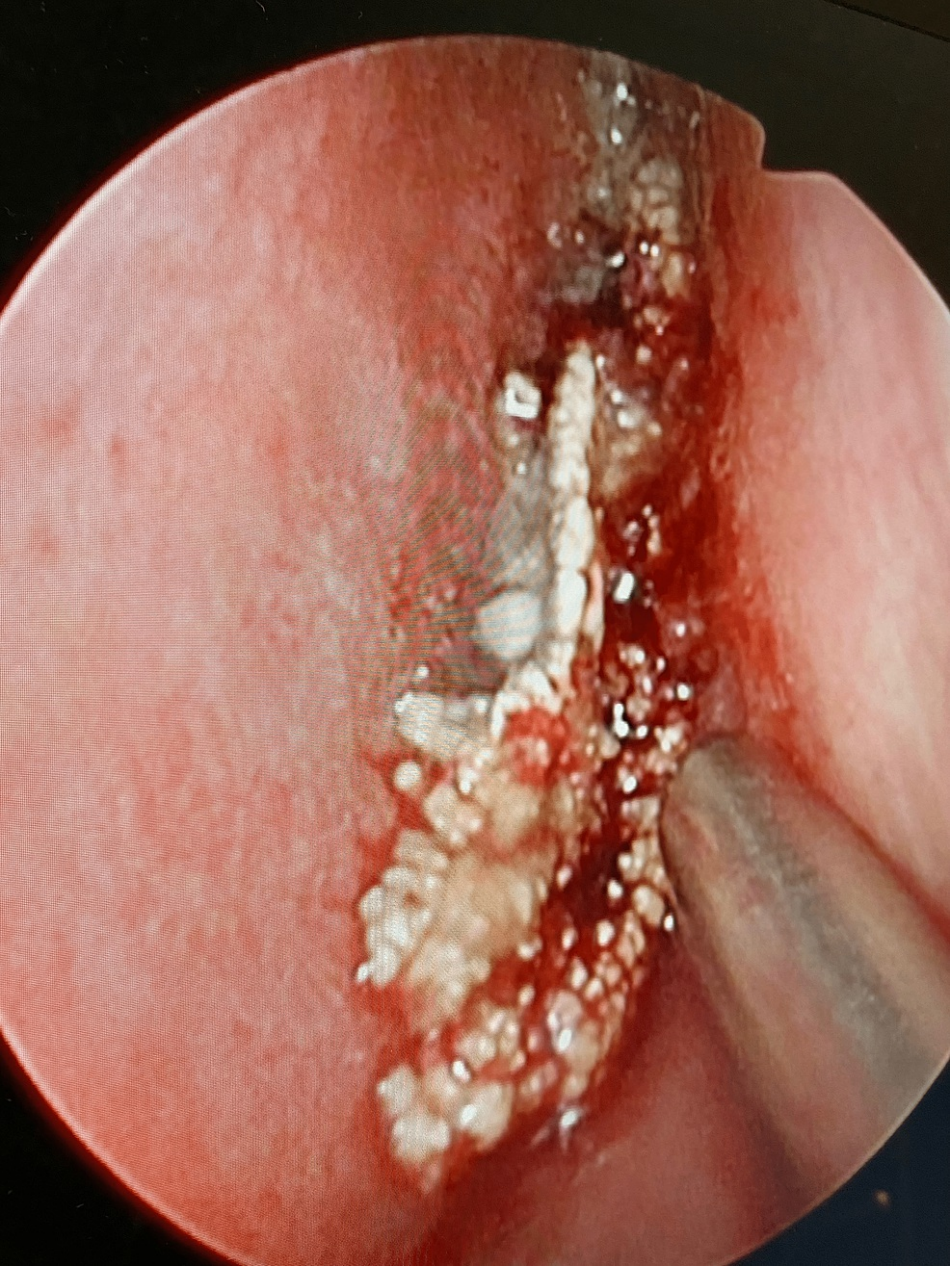
Rhinolith in the left nasal cavity

On clinical examination, using a zero-degree Hopkins rigid telescope (Karl Storz, Tuttlingen, Germany), there was a pearly white nasal mass filling the left nasal cavity, which easily bleeds, tenders upon manipulation, and completely blocks the left side, obscuring both the middle turbinate and nasopharynx (Figure 1). Examination of the right nasal cavity showed healthy nasal mucosa covering the inferior and middle turbinates without significant hypertrophy. There was no clear deviation or perforation of the nasal septum. The nasopharynx was clear.

**Figure 2:**
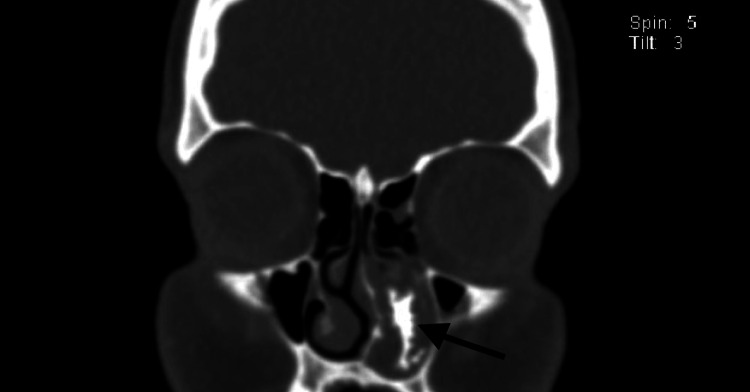
Coronal CT showing calcified iso-dense mass lesion (black arrow) measuring 2.7 × 3.4 cm that is occupying most of the left nasal cavity CT: computed tomography

Computed tomography (CT) imaging of the paranasal sinuses (Figure 2) showed a calcified iso-dense mass lesion measuring 2.7 × 3.4 cm, occupying most of the left nasal cavity, blocking the left osteomeatal complex. In addition, there was a small retention cyst and bone remodeling at the left maxillary sinus medial wall. The radiologist’s differential diagnosis included fungal sinusitis, chondrosarcoma, and inverted papilloma.

**Figure 3:**
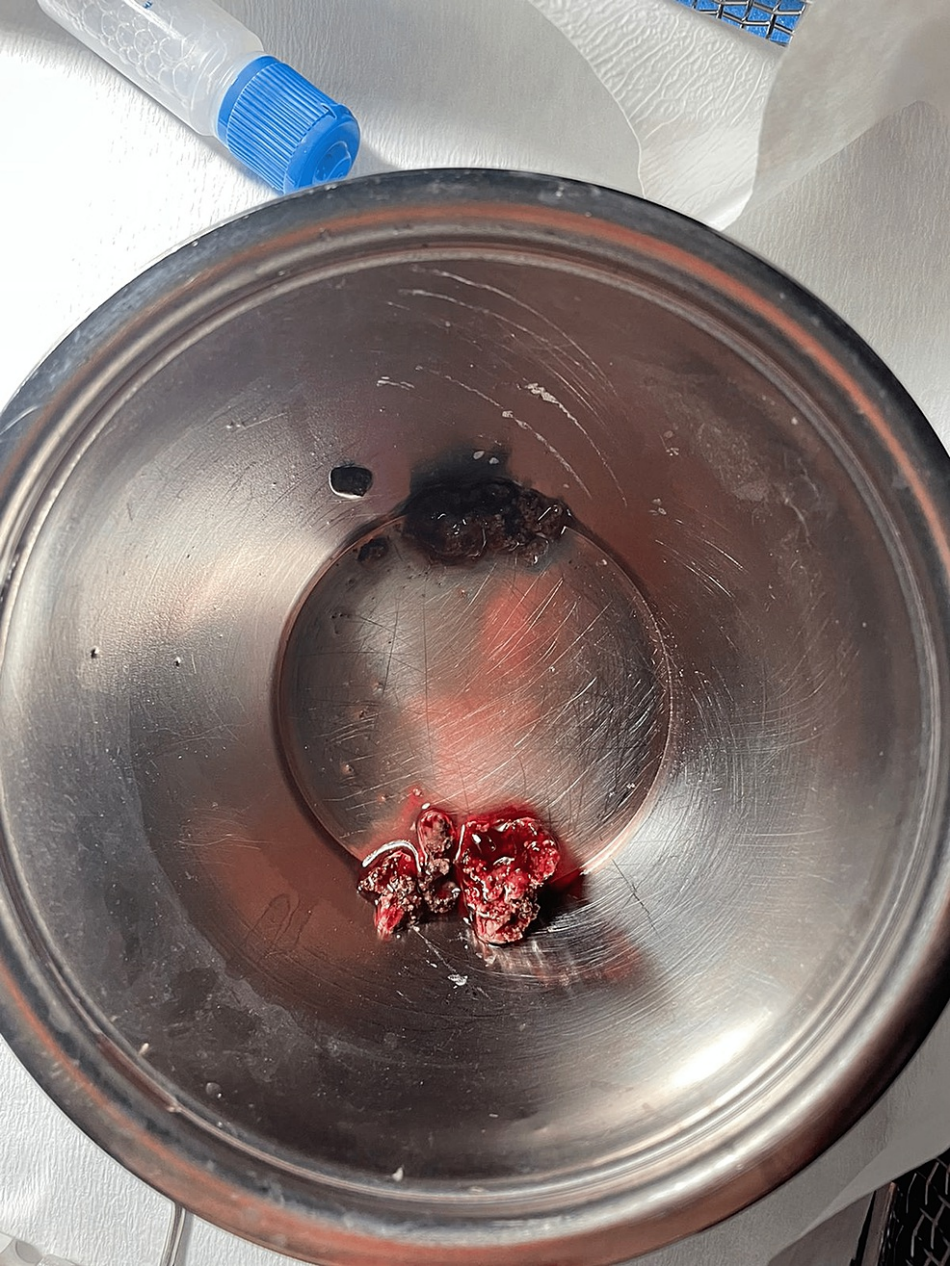
Removed rhinolith

The patient underwent endoscopic nasal examination under general anesthesia with complete removal of the left nasal mass (Figure 3). It was profusely bleeding, attached to the inferior turbinate and nasal septum. A histopathological examination revealed a fragmented inflamed respiratory epithelial and nonviable material with calcification. No fungal elements were seen and were negative for malignancy, supporting the diagnosis of rhinolith.

Two weeks postoperatively, the patient was seen in the clinic, completely asymptomatic, and nasal examination showed healthy healing nasal mucosa without postoperative adhesion or residual rhinolith.

## Discussion

Rhinolith is a concrete-like structure formed by the deposition of mineral salts, such as calcium phosphate, calcium carbonate, and magnesium, around the nidus [4,5]. This nidus could be endogenous in origin, such as teeth, clotted blood, inspissated nasal secretion, or, more commonly, exogenous due to the insertion of a foreign body inside the nose [6-8]. In our case, the nidus was exogenous as the histopathology examinations revealed a nonviable tissue.

A nasal foreign body is usually underestimated as a cause for unilateral nasal symptoms in the middle- to old-aged population. However, it can be found in any age group from an infant to 84 years old, as reported in the literature [1]. Although not clearly understood, the incidence of rhinolith is more common in the female population than in the male population [1].

Unilateral nasal obstruction, drainage, nasal and or oral malodor, headache, hyposmia, epiphora, and epistaxis are symptoms that can present in a long-standing rhinolith. However, rhinolith could be completely asymptomatic and discovered incidentally [9]. In this case study, the main symptom was a unilateral nasal blockage. The differential diagnosis includes unilateral inflammatory nasal disease with calcification, such as unilateral fungal sinusitis, calcified nasal polyps, granulomatous systemic diseases such as syphilis and tuberculosis, and benign or malignant nasal tumors with calcification, such as chondroma, osteoma, angiofibroma, inverted papilloma, chondrosarcoma, and osteosarcoma, especially in middle- to old-aged adults.

An endoscopic nasal examination is essential to help diagnose rhinolith, which usually appears like a concrete whitish mass and is commonly found between the inferior turbinate and the nasal septum [10], as in our patient. Nevertheless, rare sites have been reported in the literature [11-13].

CT scan is required to support the diagnosis; exclude other causes of unilateral nasal masses with benign radiological features such as bone remodeling and hyperostosis or suspicious features, in particular, bone destruction, invasion, and soft tissue involvement; assess associated comorbidities such as sinusitis; and rule out any complications [4]. Potential complications of rhinolith include naso-oral fistula, nasal septal perforation, palatal perforation, and social withdrawal due to nasal and oral malodor [9,14,15], and none of them were present in our case.

**Figure 4:**
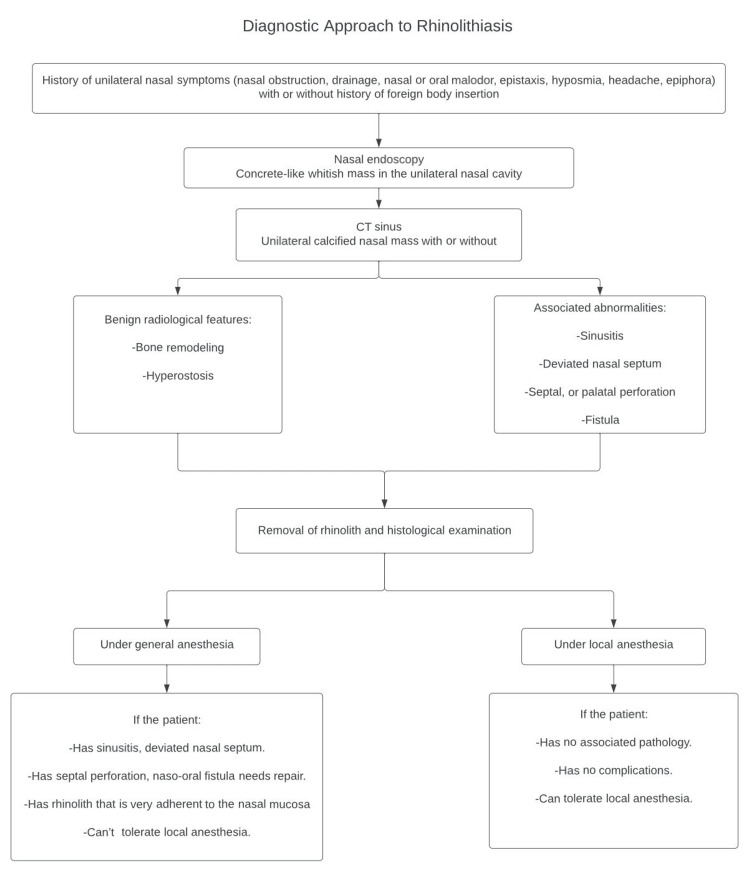
Diagnostic approach to and management of rhinolithiasis CT: computed tomography

Removal of rhinolith under general anesthesia is favorable for patients with structural abnormalities such as deviated nasal septum, with chronic sinusitis reluctant to medical treatment, as well as in the presence of complications requiring repair such as perforation or fistula. If none are present, then removal of rhinolith under local anesthesia in adults or conscious sedation in pediatrics is optional. In this report, the rhinolith is removed under general anesthesia because the patient did not tolerate the procedure under local anesthesia, as the rhinolith was very adherent to the mucosa of the nasal septum and the inferior turbinate. The diagnostic approach to and management of rhinolithiasis are presented in Figure 4.

## Conclusions

Unilateral nasal symptoms warrant an otolaryngologist for a localized disease process. Rhinolith is usually underestimated as a cause due to the rarity of the disease in young adults. Endoscopic nasal examination, imaging, and histopathological evaluation are needed to confirm the diagnosis of rhinolith.
